# Costal Cartilage Calcification in a Caucasian Population: Machine Learning Recommendations for Chest CT-guided Rhinoplasty Planning

**DOI:** 10.1055/a-2731-5948

**Published:** 2025-11-03

**Authors:** Victor S. van Dam, Diako Berzenji, Aaike S. van den Berg, Floris V. W. J. van Zijl, Andries P. Nagtegaal, Bernd Kremer, Frank R. Datema

**Affiliations:** 1 Department of Otorhinolaryngology, Erasmus Medical Center, Rotterdam, The Netherlands; 2 Department of Radiology, Erasmus Medical Center, Rotterdam, The Netherlands

**Keywords:** machine learning, costal cartilage calcification, Caucasian, rhinoplasty

## Abstract

**Introduction:**

Autologous costal cartilage calcification (CCC) can impact the course and long-term results of rhinoplasty. Preoperative information about the presence, severity, and pattern of CCC helps to assess donor site suitability and rhinoplasty planning.

**Objective:**

To use machine learning to identify a sex-specific age threshold beyond which a preoperative chest CT is likely to reveal CCC relevant for rhinoplasty planning.

**Study Design:**

Cross-sectional retrospective study of 662 Caucasian adults.

**Methods:**

Prevalence, severity, and patterns of CCC in ribs five to eight were assessed by three independent reviewers. A machine learning algorithm was used to predict the age threshold beyond which a chest CT scan is beneficial to rhinoplasty planning.

**Results:**

The prevalence of CCC in Caucasian adults was 89.6%. Nearly all individuals over the age of 50 exhibited some form of CCC. In young females CCC was more severe and prevalent in the central core of ribs five to eight compared with age-matched males.

**Conclusion:**

A chest CT is recommended in females over 23 years and males over 40 years. No data-driven recommendations regarding an upper age limit for costal cartilage use could be determined from the data.

## Introduction

Autologous costal cartilage is commonly used in rhinoplasty when septal or auricular cartilage is insufficient. 1 2 3 Typically, cartilage is harvested from the fifth to eighth ribs and the procedure is associated with low complication rates. 4 5 6 Surgical considerations include minimizing donor site morbidity (such as visible scarring, postoperative pain, and pneumothorax), anticipation to increased operative time, management of graft warping, and costal cartilage calcification (CCC). 7 8 9

**Fig. 1:**
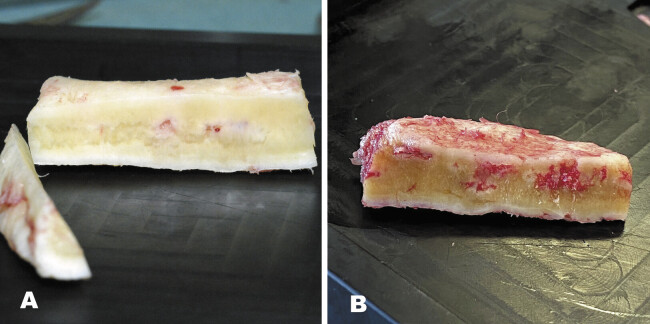
( **A** ) Costal cartilage of a 38-year-old male, showing a central pattern of calcification. ( **B** ) Costal cartilage of a 43-year-old female, showing more extensive mixed pattern of calcification and typical color change.

CCC is a physiological age-related process that can differ between males and females. 10 11 The onset of mineralization starts to occur at the end of puberty, progressively transforming the flexible cartilage into a more rigid and brittle structure ( Fig. 1A, B ). 12 Although the clinical benefit of using calcified cartilage in rhinoplasty is that it warps less, there are several disadvantages as well: it is more difficult to carve into grafts, more challenging to suture into position, and carries an increased risk of latent graft instability and resorption. 3 8 9 13

Given these limitations, some rhinoplasty surgeons recommend an upper age limit to the use of costal cartilage. 5 Others do not impose an age limit and instead manage CCC with modified carving techniques, powered instruments, or by including perichondrium to reduce graft resorption. 14 15 16 Regardless of varying expert opinions, there is consensus that intraoperative discovery of unsuitable costal cartilage should be avoided.

Preoperative assessment of presence, severity, and pattern of CCC (i.e., assessment of donor site suitability) can enhance both the course and the long-term outcomes of rhinoplasty. For this purpose, a chest CT scan is considered the gold standard. 17 Routine preoperative CT scanning is a pragmatic strategy but could raise concerns about cost-effectiveness and the judicious use of scarce radiological resources.

This study introduces a machine learning model that offers data-driven recommendations regarding a sex-specific age threshold beyond which a preoperative chest CT is highly likely to contain clinically relevant CCC. The model is built upon observational chest CT data from a large Caucasian cohort, complementing previous research on this topic, that has primarily focused on Asian populations. 18 19 Machine learning models are known to capture complex, non-linear relationships and handle high-dimensional data more effectively than traditional regression methods.

Additionally, this study investigates whether an upper age limit exists for the use of costal cartilage in rhinoplasty.

## Methods

### Study Population

The Department of Radiology provided a database comprising 2,449 consecutive non-contrast chest CT scans using Siemens Healthineers multislice CT scanners, performed between July 2019 and December 2020. All patients aged over 17 years and of Caucasian heritage were eligible for inclusion. Patients were excluded if they were of non-Caucasian heritage ( *n* = 221) and when they had a medical history that could have affected the costal cartilage: thoracic malignancy ( *n* = 589), thoracic trauma or surgery ( *n* = 343), or a combination of these conditions ( *n* = 298). Duplicate entries caused by follow-up CT scans ( *n* = 336) were removed.

### Chest CT Ratings

**Fig. 2:**
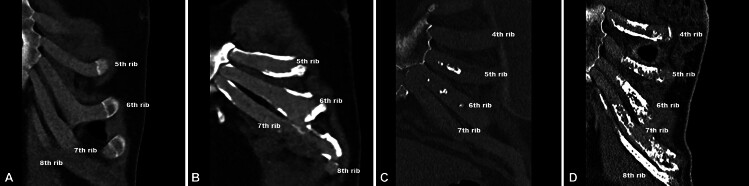
( **A** ) Coronal slide of chest CT scans demonstrating the absence of costal cartilage calcification (CCC) in ribs five to eight. ( **B** ) Coronal slide of chest CT scans demonstrating a peripheral CCC pattern, affecting <25% of cartilage volume. ( **C** ) Coronal slide of chest CT scans demonstrating a central CCC pattern, affecting <25% of cartilage volume. ( **D** ) Coronal slide of chest CT scans demonstrating a mixed CCC pattern, affecting >50% of cartilage volume.

Coronal and axial views in bone window setting were used to assess the prevalence, severity, and pattern of CCC in ribs five to eight on both sides of the thorax. In line with previous publications, the severity of CCC was defined as the ratio of visually estimated calcified volume to total cartilage volume per thorax side: 0–25, 26–50, and >50%. The pattern of CCC was categorized per rib as: none, peripheral, central, or mixed ( Fig. 2A–D ). 18 19 The scans were evaluated and rated by three independent researchers.

### Donor Site Suitability

A rib was considered a potentially unsuitable donor site in case of a *central or mixed* CCC pattern in which *>25%* of cartilage volume was affected *or* a *peripheral* CCC pattern affecting >50% of cartilage volume. Based on this criterion, individual ribs (five through eight) were assessed and categorized as either “suitable” or “unsuitable” donor site (see discussion).

A machine learning algorithm was used to predict from which age clinically relevant CCC is likely to occur (i.e., the age from which chest CT scan findings become beneficial to rhinoplasty planning). Furthermore, the upper age at which all ribs are unsuitable (i.e., the age from which a chest CT scan is no longer required) is explored.

### Statistical Analysis

To assess the consistency of the chest CT ratings, inter-observer variability was evaluated using Cohen's kappa coefficient on a random sample of 50 CT scans. A kappa value of 1.0 indicates perfect agreement. 20 To examine age- and sex-specific differences in the prevalence, pattern, and severity of CCC, the Chi-square test was applied. A *p* -value <0.05 was considered statistically significant. Statistical analyses were conducted using IBM SPSS Statistics, Version 28.0.1.1 (IBM Corp., Armonk, NY, USA).

To model the age range between which a preoperative chest CT scan is most likely to detect clinically relevant CCC, the XGBoost machine learning algorithm was used. XGBoost is an ensemble method that builds a strong predictive model through the iterative addition of gradient-boosted decision trees, where each new tree corrects the errors made by the previous ones, enhancing overall model performance. 21 The algorithm's robustness makes it particularly suited for healthcare applications, where accurate and efficient predictions are critical. The sensitivity and specificity of the model were set to 95%. For cross validation, a 5-fold strategy was used. In this approach, the dataset was split into five equal parts; in each iteration, four parts were used for training the model and one part for validation, cycling through all folds to minimize the risk of overfitting and provide an unbiased estimate of model performance.

### Ethical Considerations

The study was conducted in accordance with the Declaration of Helsinki and its later amendments up to 2024 and was approved by the Medical Ethics Review Board of the Erasmus Medical Center, Rotterdam; MEC-2020–0038.

## Results

### Population Characteristics

A total of 662 Caucasian adults were included in this study cohort, of which 342 (51.7%) were male and 320 (48.3%) were female. The median age was 51 years ± 19.0 SD (range 18–87). The categorical age distribution for males and females is separately shown in Table 1 .

**Table 1:** Age- and sex-dependent costal cartilage characteristics in paired ribs five to eight from Caucasian adults

**A. Males [** ***N*** ** = 342]**								
**Age group**		**18–30 years**	**31–40 years**	**41–50 years**	**51–60 years**	**61–70 years**	**>70 years**	**Total**
***N*** **(%)**		75 (21.9%)	50 (14.6%)	41 (12.0%)	32 (9.4%)	77 (22.5%)	67 (19.6%)	342 (100%)
**Prevalence (%)**		64.0% a	79.0%	93.9%	100%	98.1%	100%	87.9%
** Severity b**	0–25%	149 (99.3%) a	96 (96.0%)	72 (87.8%)	52 (81.3%)	131 (85.1%) a	88 (65.7%) a	588 (86.0%)
	26–50%	1 (0.7%) a	2 (2.0%)	8 (9.8%)	10 (15.6%)	19 (12.3%) a	42 (31.3%) a	82 (12.0%)
	>50%	0 (0.0%) a	2 (2.0%)	2 (2.4%)	2 (3.1%)	4 (2.6%) a	4 (3.0%) a	14 (2.0%)
** Pattern c**	None	339 (56.5%) a	176 (44.0%)	70 (21.3%)	33 (12.9%) a	48 (7.8%)	12 (2.2%) a	678 (24.8%)
	Peripheral	237 (39.5%) a	171 (42.8%)	212 (64.6%)	133 (52.0%) a	249 (40.4%)	166 (31.0%) a	1168 (42.7%)
	Mixed	13 (2.2%) a	37 (9.3%)	29 (8.8%)	61 (23.8%) a	260 (42.2%)	288 (53.7%) a	688 (25.1%)
	Central	3 (0.5%) a	4 (1.0%)	6 (1.8%)	1 (0.4%) a	14 (2.3%)	9 (1.7%) a	37 (1.4%)
	Unable to score	8 (1.3%)	12 (3.0%)	11 (3.4%)	28 (10.9%)	45 (7.3%)	61 (11.4%)	165 (6.0%)
**B. Females [** ***N*** ** = 320]**								
**Age group**		**18–30 years**	**31–40 years**	**41–50 years**	**51–60 years**	**61–70 years**	**>70 years**	**Total**
***N*** **(%)**		65 (20.3%)	44 (13.8%)	49 (15.3%)	50 (15.6%)	60 (18.8%)	52 (16.3%)	320 (100%)
**Prevalence (%)**		82.3% a	76.1%	95.9%	98.0%	99.2%	100%	91.3%
** Severity b**	0–25%	114 (87.7%) a	78 (88.6%)	82 (83.7%)	76 (76.0%)	80 (66.7%) a	40 (38.5%) a	470 (73.4%)
	26–50%	9 (6.9%) a	8 (9.1%)	14 (14.3%)	14 (14.0%)	28 (23.3%) a	24 (23.1%) a	97 (15.2%)
	>50%	7 (5.4%) a	2 (2.3%)	2 (2.0%)	10 (10.0%)	12 (10.0%) a	40 (38.5%) a	73 (11.4%)
** Pattern c**	None	195 (37,5%) a	154 (43.8%)	77 (19.6%)	59 (14.8%) a	42 (8.8%)	7 (1.7%) a	534 (20.9%)
	Peripheral	160 (30.8%) a	134 (38.1%)	151 (38.5%)	127 (31.8%) a	126 (26.3%)	45 (10.8%) a	743 (29.0%)
	Mixed	115 (22.1%) a	43 (12.2%)	126 (32.1%)	139 (34.8%) a	245 (51.1%)	317 (76.2%) a	985 (38.5%)
	Central	17 (3.3%) a	11 (3.1%)	30 (7.7%)	26 (6,5%) a	26 (5.4%)	16 (3.8%) a	126 (4.9%)
	Unable to score	33 (6.3%)	10 (2.8%)	8 (2.0%)	49 (12.3%)	41 (8.5%)	31 (7.5%)	172 (6.7%)

Abbreviations: CCC, costal cartilage calcification; *N* , number of patients.

a A statistically significant difference between males and females ( *p* < 0.05).

b Severity of CCC is shown per thorax side ( *n* = 1324).

c Pattern of CCC is defined for each individual rib ( *n* = 5,296) and presented as the percentage within each specific age category.

### Inter-observer Variability

Three independent researchers evaluated a total of 5,296 ribs. The respective kappa values of 0.92, 0.87, and 0.82 indicate a strong to almost perfect agreement between the researchers and demonstrate reliability and consistency in the data.

### Prevalence of CCC

The prevalence of CCC in ribs five to eight was 89.6% (males 87.9%; females 91.3%). As shown in Table 1 , the prevalence increases progressively with age, and above the age of 50, CCC was present in nearly all males and females. A statistically significant higher prevalence was observed in young females aged 18 to 30 years as compared with age-matched males ( *p* < 0.05). No significant differences in CCC prevalence were found between individual ribs or between the left and right sides of the thorax ( *p* = 1.00).

### Severity of CCC

The severity of CCC in ribs five to eight per thorax side ( *n* = 1324) is shown in Table 1 . Independent of age and sex, a 0 to 25% severity was observed in most adults (79.9%), followed by 26 to 50% (13.4%) and >50% (6.6%). Severity increases progressively with age. A statistically significant higher severity was observed in young females aged 18 to 30 years as compared with age-matched males ( *p* < 0.05); a similar finding was observed in females over the age of 60.

### Patterns of CCC

In Table 1 , the patterns of CCC are shown for each separate rib, distributed in age categories for males and females separately. A peripheral pattern of calcification was present in 1,911 ribs (36.1%), a central pattern in 163 ribs (3.1%), and a mixed pattern in 1,673 ribs (31.6%). In 337 cases, the 8th rib (6.4%) was not clearly depicted, making it impossible to define a reliable pattern. Overall, the central calcification pattern is more frequently observed in females, and a peripheral pattern more frequently in males. Eventually, as age progresses, the mixed pattern becomes predominant in both sexes. It is notable that among young females aged 18 to 30 years, central and mixed CCC was statistically significantly more prevalent than in age-matched males ( *p* < 0.05); a similar finding was observed in females over the age of 50.

### Machine Learning Model—When to Perform a Chest CT Scan

**Fig. 3:**
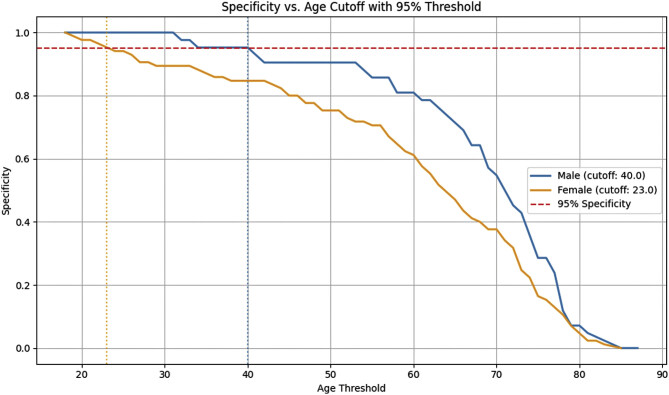
Graphical presentation of XGBoost model output using 95% specificity, showing the age cutoff from which a chest CT scan starts to show clinically relevant costal cartilage calcification (CCC) findings for rhinoplasty planning. In this example the age cutoff for males is 40.0 years and for females 23.0 years. Explore age threshold changes per sex when choosing a lower specificity (Y-axis).

Based on a specificity of 95%, the age at which a chest CT scan reveals clinically relevant findings for rhinoplasty planning is 23 years for females and 40 years for males ( Fig. 3 ). The lower threshold for females aligns with the statistically significant differences in prevalence, pattern, and severity compared with age-matched males. The AUC for the corresponding age groups 18–30 and 31–40 was 0.74 and 0.71, respectively, indicating good model performance.

The age at which CCC renders all ribs unsuitable as a donor site was 81 years for females and 82 years for males. Given the low probability of patients undergoing rhinoplasty at such advanced ages, the clinical relevance of an upper age limit for the use of autologous rib cartilage seems debatable. However, the AUC value of 0.49 for the corresponding age category (>70 years) does not allow solid conclusions.

## Discussion

This cross-sectional observational chest CT study investigated the prevalence, patterns, and severity of CCC in the 5th to 8th ribs of Caucasian adults, with a focus on implications for rhinoplasty. Using previously established methodologies, we aim to complement existing literature that has predominantly focused on Asian populations or cadaveric specimens with variable sample sizes. 5 10 18 19

We observed a CCC prevalence of 89.6% in Caucasian adults, which is slightly lower than the 96.7% reported by Hawellek et al 5 in a cadaveric series of unspecified demographic origin, yet substantially higher than the 50.8% reported by Sunwoo et al, 19 who used a comparable imaging methodology. This discrepancy may be explained by the absence of the 5th rib in their analysis or the use of 3-mm image slice thickness, which could have limited sensitivity to detect CCC. Alternatively, despite our defined exclusion criteria, underlying medical conditions may have affected CCC in our cohort. A retrospective review of medical records revealed that 104 patients (15.7%) had auto-immune diseases treated with immunosuppressive medications. Chronic corticosteroid use is associated with an accelerated CCC process. 22 23 However, in a separate analysis, no statistically significant difference in CCC prevalence was observed between these patients and the rest of the cohort. We also found no statistically significant differences in CCC prevalence between individual ribs five to eight on either side, while in two separate Asian cohorts the 8th rib was identified as the least calcified. 18 19 These findings suggest that ethnic variations may influence CCC prevalence, highlighting the need for further comparative studies across different populations.

Consistent with the literature our study demonstrated age-related increase in CCC prevalence. Table 1 displays that nearly all males and females over the age of 50 exhibit some degree of calcification. Importantly, a significantly higher prevalence was observed in young females aged 18 to 30 years (82.3%) compared with age-matched males (64.0%). Furthermore, within this age group more severe CCC and more central and mixed patterns were observed in females, whereas peripheral CCC was more common in males. These findings highlight the need for careful CCC assessment in younger rhinoplasty patients, especially females.

An important clinical question derived from these results is at what age chest CT imaging becomes beneficial for preoperative planning of autologous rib rhinoplasty. A practical limitation to answering this question is the absence of a formal grading system or globally accepted definition for costal cartilage suitability—the outcome variable of our predictive model. To address this, we combined CCC conditions most likely affecting graft harvest and surgical outcomes, particularly in cases requiring long, straight, and strong grafts, cut from the central core of the rib. We considered a rib as a potentially unsuitable donor site in case of *central or mixed* CCC pattern involving *more than 25%* of cartilage volume, or a *peripheral* CCC pattern affecting *more than 50* %. This definition applied to 105 out of 662 (15.9%) adults. The XGBoost machine learning algorithm demonstrated a strong predictive performance in the younger age categories. Based on a 95% specificity threshold, we recommend chest CT scans for females over 23 years and males over 40 years. The lower threshold for females corresponds to the statistically significant sex differences in CCC prevalence, severity, and pattern. The XGBoost model underwent extensive cross validation. An external validation study comparing preoperative CT findings with intraoperative observations is currently being performed to confirm the accuracy of our results.

We anticipate that there will be colleagues who are not convinced that our model is clinically beneficial. Some surgeons never perform a chest CT scan, because they do not believe in the disadvantages of CCC and a subsequent age threshold, especially since the popularization of powered instruments and alternative cartilage cutting techniques. Other surgeons always perform a chest CT, but this seems unnecessary, not cost-effective, and not evidence based. A third group relies on intraoperative ultrasonography or transcutaneous needle palpation to identify calcified zones and donor site selection. 24 Despite this variety in clinical approaches and opinions, we feel that data-driven recommendations are beneficial to rhinoplasty planning and the judicious use of scarce resources.

Regarding the upper age limit for costal cartilage use in rhinoplasty, an online poll by the Evidence in Rhinoplasty Research Group (EBRRG) found that most experts (37%) consider 40 to 70 years appropriate. This aligns with our finding that nearly all adults over 50 exhibit some degree of CCC. However, the mere radiologic presence of CCC does not necessarily indicate clinical relevance to rhinoplasty. Using machine learning, we estimated that all ribs become unsuitable at 81 years for females and 82 for males. Given the low probability of patients undergoing rhinoplasty at such advanced ages, the clinical relevance of these limits is uncertain. Moreover, the model's poor performance (AUC = 0.49) for patients over 70 years does not allow us to make evidenced-based recommendations.

## Conclusion

CCC is an age- and sex-dependent physiological process. In a large cohort of Caucasian adults, the prevalence of CCC was 89.6%. In the context of rhinoplasty, it is important to recognize that moderate to severe CCC affecting the central core of ribs five to eight is more prevalent in young females as compared with age-matched males. Based on a machine learning algorithm, preoperative chest CT imaging to improve donor site assessment and selection is recommended for females over 23 years and males over 40 years. No data-driven recommendations regarding an upper age limit to the use of autologous costal cartilage could be established using machine learning.
